# Susceptibility of *Actinobacillus pleuropneumoniae*, *Pasteurella multocida* and *Streptococcus suis* Isolated from Pigs in Hungary between 2018 and 2021

**DOI:** 10.3390/antibiotics12081298

**Published:** 2023-08-08

**Authors:** Zoltán Somogyi, Patrik Mag, Réka Simon, Ádám Kerek, László Makrai, Imre Biksi, Ákos Jerzsele

**Affiliations:** 1 Department of Pharmacology and Toxicology, University of Veterinary Medicine, István Str. 2., H-1078 Budapest, Hungary; mag.patrik@univet.hu (P.M.); simon.reka@student.univet.hu (R.S.); kerek.adam@univet.hu (Á.K.); jerzsele.akos@univet.hu (Á.J.); 2 National Laboratory of Infectious Animal Diseases, Antimicrobial Resistance, Veterinary Public Health and Food Chain Safety, University of Veterinary Medicine, H-1078 Budapest, Hungary; 3 Department of Microbiology and Infectious Diseases, University of Veterinary Medicine, István Str. 2., H-1078 Budapest, Hungary; 4 Department of Pathology, University of Veterinary Medicine, István Str. 2., H-1078 Budapest, Hungary; biksi.imre@univet.hu; 5 SCG Diagnostics Ltd., HU-2437 Délegyháza, Hungary

**Keywords:** *Actinobacillus pleuropneumoniae*, *Pasteurella multocida*, *Streptococcus suis*, MIC, antibacterial agents, swine

## Abstract

Porcine respiratory disease complex (PRDC) has been a major animal health, welfare, and economic problem in Hungary; therefore, great emphasis should be put on both the prevention and control of this complex disease. As antibacterial agents are effective tools for control, antibiotic susceptibility testing is indispensable for the proper implementation of antibacterial therapy and to prevent the spread of resistance. The best method for this is to determine the minimum inhibitory concentration (MIC) by the broth microdilution method. In our study, we measured the MIC values of 164 *Actinobacillus pleuropneumoniae*, 65 *Pasteurella multocida*, and 118 *Streptococcus suis* isolates isolated from clinical cases against the following antibacterial agents: amoxicillin, ceftiofur, cefquinome, oxytetracycline, doxycycline, tylosin, tilmicosin, tylvalosin, tulathromycin, lincomycin, tiamulin, florfenicol, colistin, enrofloxacin, and sulfamethoxazole-trimethoprim. Outstanding efficacy against *A. pleuropneumoniae* isolates was observed with ceftiofur (100%) and tulathromycin (100%), while high levels of resistance were observed against cefquinome (92.7%) and sulfamethoxazole-trimethoprim (90.8%). Ceftiofur (98.4%), enrofloxacin (100%), florfenicol (100%), and tulathromycin (100%) were found to be highly effective against *P. multocida* isolates, while 100% resistance was detected against the sulfamethoxazole-trimethoprim combination. For the *S. suis* isolates, only ceftiofur (100%) was not found to be resistant, while the highest rate of resistance was observed against the sulfamethoxazole-trimethoprim combination (94.3%). An increasing number of studies report multi-resistant strains of all three pathogens, making their monitoring a high priority for animal and public health.

## 1. Introduction

Porcine Respiratory Disease Complex (PRDC) is most commonly found in weaning and weaning-to-finishing pigs and is a complex disease, i.e., in most cases caused by multiple pathogens or facultative pathogens. The primary pathogenic agents of PRDC are *Mycoplasma hyopneumoniae*, *Actinobacillus pleuropneumoniae*, and *Bordetella bronchiseptica*, as well as viruses causing various respiratory pathologies [1,2]. *Pasteurella multocida*, *Streptococcus suis*, *Glässerella parasuis*, and *Salmonella Choleraesuis* are usually considered secondary pathogens [3]. These primary and secondary pathogens together form PRDC, whose severity gradually increases until it is out of control [4,5,6].

The continuous monitoring of antibacterial agents is a very important task, as it is not only of animal health, animal welfare, and economic importance but also of paramount public health importance. The European Committee on Antimicrobial Susceptibility Testing [7] plays an important role in this, not only by testing pathogens coming from public health but also by testing pathogens of animal origin. This includes the antibiotic susceptibility of bacteria involved in the development of PRDC. There are further proposals for the establishment of an EU-wide antimicrobial resistance (AMR) monitoring system [8,9].

Determining the minimum inhibitory concentration (MIC) of PRDC-causing bacteria not only allows us to measure the prevalence of different resistant strains but also provides a quantitative result that helps determine the exact antibacterial therapy. The MIC value can also be used to perform pharmacokinetic/pharmacodynamic analysis, which is important in determining the dose of antibacterial agents. The dose helps select the appropriate route of drug administration and the frequency of antibacterial agent use, i.e., the time interval between two administrations [10,11]. There have been fewer studies on the antibiotic susceptibility of PRDC pathogens in the last five years than in the early 2010s, which further strengthens the need for this [12].

In the present study, we investigated the antibiotic susceptibility of three bacterial species most frequently responsible for respiratory tract infections in pigs in Hungary. *Actinobacillus pleuropneumoniae*, *Pasteurella multocida*, and *Streptococcus suis* strains were evaluated for their susceptibility to amoxicillin, ceftiofur, cefquinome, oxytetracycline, doxycycline, tylosin, tilmicosin, tylvalosin, tulathromycin, lincomycin, tiamulin, florfenicol, colistin, enrofloxacin, and sulfamethoxazole-trimethoprim.

Amoxicillin, ceftiofur, and cefquinome belong to the beta-lactam group of antibiotics that are used extensively in pig medicine. According to the EMA classification, amoxicillin is one of the antimicrobials that should be the first choice in veterinary medicine, thus reducing the use of more important public health agents such as ceftiofur and cefquinome [13,14,15,16,17].

Oxytetracycline, doxycycline, and sulfamethoxazole-trimethoprim, like amoxicillin, should be prioritised in animal health. Unfortunately, antimicrobial resistance to these agents is the highest. For this reason, it is of paramount importance to assess the antibiotic susceptibility of pathogenic bacteria prior to the use of these products [14,18,19,20,21].

Macrolides (tylosin, tilmicosin, tylvalosin, and tulathromycin), lincomycin, tiamulin, and florfenicol are some of the most important agents in the treatment of respiratory tract infections in pigs, so the assessment of resistance to them is very important in pig medicine. They are also of public health importance, as cross-resistance between macrolides and oxazolidinones (linezolid), which are important for public health, can occur [14,18,22,23,24,25,26,27,28,29,30,31].

Colistin is a less important antibacterial agent in respiratory diseases of pigs, but its public health importance is paramount, so it is important to confirm or exclude the presence of resistance to it in all Gram-negative pathogens [14,32,33,34].

The susceptibility of bacteria to enrofloxacin is important at both the public health and pig health levels, as it is still commonly used in most countries, despite its public health importance being as high as that of colistin [14,35,36,37,38,39,40].

## 2. Results

### 2.1. Susceptibility of Actinobacillus pleuropneumoniae Isolates

The susceptibility of 164 *A. pleuropneumoniae* clinical isolates collected by us was tested against 15 antibacterial agents. The clinical breakpoint and epidemiological cut-off value for MIC set by the Clinical and Laboratory Standards Institute (CLSI) or the European Committee on Antimicrobial Susceptibility Testing (EUCAST) are available for the following antibacterial agents: amoxicillin, ceftiofur, cefquinome, oxytetracycline, tilmicosin, tulathromycin, tiamulin, florfenicol, enrofloxacin, and sulfamethoxazole-trimethoprim [7,41]. Thus, sensitivity was only determined for these agents. High susceptibility to ceftiofur (100%), tilmicosin (95.7%), tulathromycin (100%), and florfenicol (98.8%) was observed for the tested isolates [41], with low MIC_90_ values (0.125 µg/mL, 8 µg/mL, 16 µg/mL, and 4 µg/mL, respectively). In contrast, a lower percentage of the tested isolates were sensitive to amoxicillin (64.0%), oxytetracycline (44.4%), tiamulin (70.5%), and enrofloxacin (47.5%) [41]. For amoxicillin, oxytetracycline, and tiamulin, MIC_90_ values of 64 µg/mL were determined in all cases, while for enrofloxacin, the MIC_90_ value was 4 µg/mL. Only 7.3% and 9.8% of the isolates were found to be sensitive to cefquinome and sulfamethoxazole-trimethoprim, respectively, taking into account the epidemiological cut-off values set by EUCAST [7]. These agents were associated with MIC_90_ values of 0.25 µg/mL and 64 µg/mL. For tylvalosin, the MIC values were 64 µg/mL for both MIC_50_ and MIC_90_. For tylosin and lincomycin, similar MIC values were observed (MIC_50_ 32 µg/mL and MIC_90_ 64 µg/mL). For the other drugs, MIC values were the following: for doxycycline, MIC_50_ was 1 µg/mL, MIC_90_ was 16 µg/mL, and for colistin, MIC_50_ was 0.015 µg/mL, and MIC_90_ was 1 µg/mL. The susceptibility of *A. pleuropneumoniae* strains to the tested drugs is summarised in Table 1 and Table 2.

**Figure 1:**
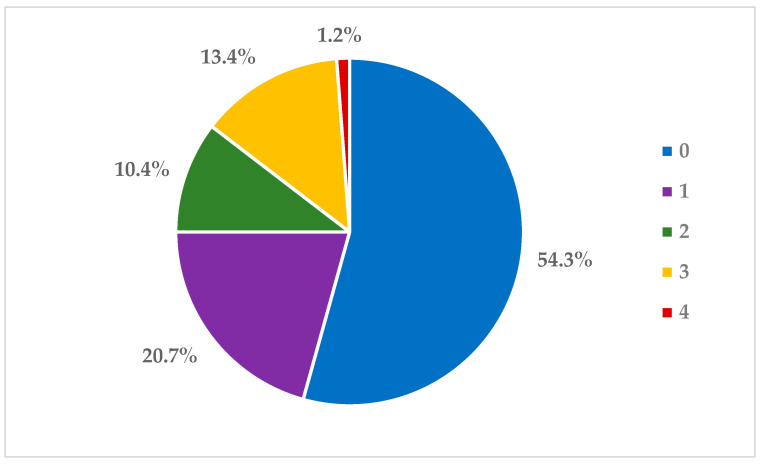
Percentage distribution of isolates according to the number of antimicrobials to which they show resistance.

Comparing the susceptibility of the tested isolates with the clinical breakpoints defined by CLSI, 89 isolates (54.3%) did not show resistance to any antimicrobial agent. A total of 34 isolates (20.7%) showed resistance to only one antibiotic, while 17 isolates (10.4%) showed resistance to two antibiotics. A total of twenty-four isolates proved to be multidrug resistant (MDR), of which twenty-two isolates (13.4%) showed resistance to three antibiotics and two isolates (1.2%) showed resistance to four antibiotics (Figure 1).

In total, 13 AMR profiles were identified among *A. pleuropneumoniae* isolates. Of these, five profiles were identified where the isolates showed resistance to three or more antibiotics (MDR profiles). A total of 24 (14.6%) isolates belonged to these MDR profiles. Half of these isolates were in one profile (profile 1). Of the other half of the isolates, eight isolates were in profile two, two isolates were in profile three, and one isolate was in both profiles four and five (Table 3).

### 2.2. Susceptibility of Pasteurella multocida Isolates

The 65 *P. multocida* clinical isolates were tested for 12 antibacterial agents. Clinical breakpoints or epidemiological cut-off values defined by CLSI or EUCAST are available for most of the agents we tested [7,41]. Exceptions are tylosin and lincomycin, for which identical MIC_50_ (16 µg/mL) and MIC_90_ (32 µg/mL) values were determined, and colistin, for which lower MIC values were found compared to the two antibacterial agents (MIC_50_ 0.03 µg/mL and MIC_90_ 0.06 µg/mL). Compared to the CLSI clinical breakpoints and the EUCAST epidemiological cut-off values, doxycycline was found to be the least effective, with only 69.2% of *P. multocida* isolates being susceptible and a MIC_90_ of 2 µg/mL. For ceftiofur, tilmicosin, and tiamulin, >90% of the isolates were sensitive (MIC_90_ 0.125 µg/mL, 8 µg/mL, and 16 µg/mL), and all isolates were sensitive for amoxicillin, tulathromycin, florfenicol, and enrofloxacin (MIC_90_ 1 µg/mL, 0.5 µg/mL, 1 µg/mL, and 0.06 µg/mL). The susceptibility of *P. multocida* isolates to the tested agents is summarised in Table 4 and Table 5.

### 2.3. Susceptibility of Streptococcus suis Isolates

The susceptibility of 118 clinical isolates of *S. suis* to 11 antibacterial agents was determined. One hundred percent of the isolates were sensitive to ceftiofur, with a MIC_90_ of 1 µg/mL. For florfenicol, 75.4% of isolates (MIC_90_ 8 µg/mL) and for enrofloxacin, 82.9% of isolates (MIC_90_ 8 µg/mL) were sensitive. The *S. suis* isolates tested were highly resistant to the combination sulfamethoxazole-trimethoprim, with only 5.7% being sensitive and a MIC_90_ of 256 µg/mL. The MIC values of the other tested substances were variable. Amoxicillin had the lowest MIC value (MIC_50_ 0.06 µg/mL and MIC_90_ 4 µg/mL). Doxycycline had a MIC_50_ of 8 µg/mL and a MIC_90_ of 32 µg/mL. The MIC_50_ values for tylosin, tilmicosin, tulathromycin, and lincomycin differed (32 µg/mL, 8 µg/mL, 2 µg/mL, and 32 µg/mL, respectively), but their MIC_90_ values were the same (128 µg/mL). The sensitivity of the isolates to the tested drugs is summarised in Table 6 and Table 7.

## 3. Discussion

The continuous monitoring of antibacterial agents is an extremely important task for veterinarians, as it has not only a veterinary and economic purpose but is also beneficial for public health. Continuous monitoring of the susceptibility to antibacterial products used in food-producing farm animals helps us optimise therapeutic management and inhibit the spread of antimicrobial resistance. In order to reduce resistant strains of bacteria, Hungary has already tightened the conditions of antibiotic use [42], based on the European Union (EU) Directive 2001/82/EC [43], and from 8 January 2022, the stricter use of antibiotics has been regulated at EU level [44]. By monitoring the resistance profile of bacterial strains on livestock farms, we can successfully determine the antibacterial agents of choice at the herd level, further reducing the chance of selection of resistant strains.

In order to set clinical breakpoints at the national level to assist veterinarians working in livestock farms, it would be necessary to carry out harmonised susceptibility testing using the same methodology to determine the susceptibility of bacteria in different farms. The development of such a test method and the creation of a central database would be a valuable addition to national veterinary practice and, if harmonised at an international level, would open up new ways of combating resistant strains of bacteria. To implement the previous proposal, our research group applied for and won a grant to establish a National Laboratory for Animal Infectious Diseases, Antimicrobial Resistance, Veterinary Public Health, and Food Chain Safety, which will allow this work to be carried out.

### 3.1. Susceptibility of Actinobacillus pleuropneumoniae Isolates

The isolates of *A. pleuropneumoniae* we tested were 100% sensitive to ceftiofur and tulathromycin, which is in agreement with the previous literature [6,8,45,46,47,48]. However, 92.7% of the isolates we tested were resistant to cefquinome. Cefquinome is a frequently used antibacterial agent in the pig farming industry in Hungary. Unfortunately, the use of cefquinome may increase the chance of the selection of bacteria resistant to beta-lactam antibiotics [14,16,17]. Based on our results, it would be advisable to greatly reduce its use in animal health. This would presumably have positive results, both in animal health and in public health. To florfenicol, 98.8% of isolates were sensitive, and to tilmicosin, 95.7% of isolates were sensitive; the results are in agreement with the data previously published by others [6,8,45,46,47,48,49]. However, it was observed that while in previous studies more than 95% of the isolates were sensitive to tiamulin [6,8,46,48], only 70.5% of the isolates tested in our study were sensitive to this antibiotic. To amoxicillin, 64.0% of the isolates were sensitive, which is moderate compared to that observed by Vilaró et al. (72.2%) [48]. There is a divergent picture in the literature on the sensitivity of *A. pleuropneumoniae* isolates to tetracyclines. While in a Danish study by Holmer et al., 92.4% of isolates were sensitive to the antibacterial agent [8], only 76% of isolates were sensitive in a Czech Republic study by Kucerova et al. [46], and 70% of isolates were sensitive in a European monitoring programme by El Garch et al. [6]. In the Spanish survey by Gutiérrez-Martín et al., only 26.2% of the strains were sensitive to tetracyclines [49]. In the North American survey by Portis et al., less than 7.4% of the isolates were sensitive to tetracyclines [45], while in the North American survey by Sweeney et al., all strains were resistant [47]. A total of 44.4% of our isolates were sensitive, which is in line with what has been described previously in Central and Eastern Europe. For enrofloxacin, in most cases, more than 95% of strains showed sensitivity [6,45,47], with only Vilaró et al. describing a lower sensitivity (72.2%) [48]. In comparison, 47.5% of the isolates we collected were sensitive, which is strikingly low compared to previous results. The case is even more severe for the sulfamethoxazole-trimethoprim combination, where 90.8% of the isolates were resistant, while the same value in the literature is below 12% [6,48].

For doxycycline, our MIC values (MIC_50_ 1 µg/mL and MIC_90_ 16 µg/mL) were higher than previously reported by Vilaró et al. (MIC_50_ 0.5 µg/mL and MIC_90_ 4 µg/mL) [48] and Yuan et al. (MIC_90_ 8 µg/mL) [50]. The same can be said for lincomycin, where the MIC_50_ and MIC_90_ of the isolates we collected were 32 µg/mL and 64 µg/mL, while values of 16 µg/mL and 32 µg/mL were reported in the literature for the same values [6,51]. For tylosin, the MIC_90_ of our isolates was the same as that reported in the El Garch et al. study (64 µg/mL) [6]. For colistin, MIC values were lower (MIC_50_ 0.015 µg/mL and MIC_90_ 1 µg/mL) than those reported in the Swiss study by Matter et al. (MIC_50_ 1 µg/mL and MIC_90_ 2 µg/mL) [52].

Based on these data, the most effective antibacterial agents for *A. pleuropneumoniae* infection will be ceftiofur and tulathromycin. Florfenicol and tilmicosin also have outstanding efficacy against the bacteria. Nationally, enrofloxacin and tiamulin have been shown to be less effective, so these antibiotics should only be used after sensitivity testing. The moderate susceptibility of amoxicillin and oxytetracycline is in line with the literature, while cefquinome and sulfamethoxazole-trimethoprim have been found to have high resistance in the national context and should be used only in exceptional cases after sensitivity testing.

### 3.2. Susceptibility of Pasteurella multocida Isolates

The *P. multocida* isolates we tested were all sensitive to amoxicillin, enrofloxacin, florfenicol, and tulathromycin. Similar results can be found in the literature for all four antibacterial agents [6,47,48,53], but less pronounced susceptibilities have been reported for enrofloxacin and florfenicol (93.1% [54] and 81.5% [53]). Our result for ceftiofur, where 98.4% of isolates were sensitive, is similar to that described by Oh et al. [53], but most of the literature data suggest that 100% of the isolates tested are usually sensitive to ceftiofur [6,45,47,48,54]. To tilmicosin, 95.2% of the isolates were sensitive, which is in agreement with the results reported in the literature [6,45,47,48,53], while to tiamulin, 97.7% of the isolates were sensitive, which is higher than that reported by Vilaró et al. (60.8%) [48]. For amoxicillin (100.0% of isolates) and for doxycycline (69.2% of isolates), we found a higher sensitivity than Vilaró et al. (96.2% and 51.5%, respectively) [48]. For sulfamethoxazole-trimethoprim, El Garch et al. [6] and Oh et al. [53] reported a sensitivity above 90% for the tested isolates, whereas Vilaró et al. [48] reported a sensitivity of only 74.7%. In contrast, the isolates collected by us showed 100% resistance to this substance.

For tylosin, our MIC_90_ value was 32 µg/mL, which is in agreement with that reported by Oh et al. [53] and lower than that reported by El Garch et al. [6] (64 µg/mL). In relation to lincomycin, the MIC_90_ value for the isolates collected by us was 32 µg/mL, which is the same as described by El Garch et al. [6], but higher than that published by Cuevas et al. (16 µg/mL) [54].

These suggest that in the case of *P. multocida* infection, all isolates will show sensitivity to ceftiofur and tulathromycin, similar to that of *A. pleuropneumoniae* infection, as well as to amoxicillin, enrofloxacin, and florfenicol. Approximate sensitivities were also observed for tilmicosin and tiamulin, which are higher for the latter agent than previously reported in the literature. Doxycycline was only effective in half of the isolates, so its empirical use should be avoided, while in the national context, the combination sulfamethoxazole-trimethoprim showed complete resistance to the isolates we tested, so its use should only be considered after a sensitivity study and only if the results are favourable.

### 3.3. Susceptibility of Streptococcus suis Isolates

In our study, 100% of *S. suis* isolates were susceptible to ceftiofur, which is in agreement with the data published in the literature [6,8,45,47,55,56,57], but it is worth noting that in most cases, researchers have already reported a few percent resistance, compared to *A. pleuropneumoniae* and *P. multocida* isolates, where 100% of isolates were sensitive in most published results. In the case of enrofloxacin and florfenicol, only 82.9% and 75.4% of our isolates were sensitive, respectively, which is lower than that reported in the literature. A sensitivity of over 90% of isolates for florfenicol and over 95% for enrofloxacin has been previously described by researchers [6,8,45,47,55,56,57]. For sulfamethoxazole-trimethoprim, in most cases more than 90% of the isolates were sensitive [8,55,57], but in the study by Hernandez et al., only 79% of the isolates were sensitive [56]. In contrast, 94.3% of the isolates we studied were resistant to potentiated sulfonamide.

In the case of amoxicillin, the MIC_90_ of the isolates we tested was 4 µg/mL, whereas previously in the literature a value of 0.06 µg/mL or less was described [6,56]. Doxycycline also had a higher MIC_90_ (32 µg/mL) than previously described (16 µg/mL) [56]. The MIC_90_ value obtained for tylosin, tilmicosin, tulathromycin, and lincomycin was consistently 128 µg/mL, which is in agreement with results published by others [6,8,45,47,55,56,57], but the MIC_90_ value obtained for tiamulin (128 µg/mL) was higher than previously described (16 µg/mL and 32 µg/mL) [6,56].

In conclusion, ceftiofur will be the best choice against *S. suis* isolates in the national context, while enrofloxacin and florfenicol, which have shown excellent efficacy in the literature, show more moderate efficacy among the national isolates. In the case of sulfamethoxazole-trimethoprim, outstanding resistance among national isolates was observed, and this antibacterial agent can only be used after sensitivity testing.

## 4. Materials and Methods

### 4.1. Laboratory Participants and Isolate Characterisation

From clinical cases collected in Hungary between 2018 and 2021, isolates of *A. pleuropneumoniae*, *P. multocida*, and *S. suis* were obtained from the Department of Epidemiology and Microbiology, University of Veterinary Medicine Budapest, and SCG Diagnostics Ltd. (East Hartford, CT, USA).

The isolates were collected and processed continuously throughout the study. Identification to species level was carried out based on their culture, morphological characteristics, and biochemical characteristics using commercially available kits (such as API Microbial Identification Kits, bioMerieux, Durham, NC, USA). Each isolate was stored in a mixture of 800 µL of tryptone soy broth (PharmaBio^®^ Tryptone Soy Broth, Biolab Zrt., Budapest, Hungary) and 200 µL of sterile glycerol (Glycerol 87% P.A., Lach-Ner, Ltd., Neratovice, Czech Republic) at −80 °C until antimicrobial susceptibility testing was performed.

### 4.2. Determination of Minimal Inhibitory Concentration Values

The in vitro susceptibility tests were performed at the Department of Pharmacology and Toxicology, University of Veterinary Medicine Budapest. All MICs were performed in tryptone soy broth (PharmaBio^®^ Triptone Soy Broth, Biolab Zrt., Budapest, Hungary), and for *A. pleuropneumoniae* strain I, NAD (β NAD, Biolab Zrt., Budapest, Hungary) supplementation was used, following the Clinical and Laboratory Standards Institute (CLSI) specifications, so that our results are reproducible and internationally comparable with those of other laboratories. For each isolate, the final concentration of bacteria in the broth dilution was 5 × 10^5^ colony-forming units per mL (CFU/mL). Soup dilutions were performed on 96-well microplates (96-well BRANDplates—F—pureGrade S, VWR International, Radnor, PA, USA). The 96-well microplate was used to prepare a two-based dilution series of the following antibacterial agents: amoxicillin, ceftiofur, cefquinome, oxytetracycline, doxycycline, tylosin, tilmicosin, tylvalosin, tulathromycin, lincomycin, tiamulin, florfenicol, colistin, enrofloxacin, and sulfamethoxazole-trimethoprim. The concentration range of antibacterial agents was selected based on quality control principles and clinical limits [7,41].

### 4.3. Number of Isolates Tested per Antibiotic

In this study, we tested the susceptibility of 164 clinical isolates of *A. pleuropneumoniae*, 65 clinical isolates of *P. multocida*, and 118 clinical isolates of *S. suis*, for a total of 15 substances. The number of isolates tested per agent is summarised in Table 8.

### 4.4. Data Analysis

MIC_50_ and MIC_90_ values were determined for all clinical isolates. Where possible, these values were compared with the clinical breakpoints and epidemiological cut-off values set by CLSI and EUCAST. The percentage of the number of susceptible and resistant strains expressed as a percentage was determined (Table 1 and Table 3, Table 4, Table 5, Table 6 and Table 7).

Multidrug-resistant bacteria were defined as having acquired non-susceptibility to at least one agent in three or more antimicrobial categories [58]. For the *A. pleuropneumoniae* isolates, AMR profiles of the MDR bacteria were performed against those agents for which CLSI clinical breakpoints were available [41] (Table 2, Figure 1). For *P. multocida* and *S. suis*, CLSI breakpoints were available for only a few antimicrobial agents, and outstanding efficacy was observed against the isolates for these agents, so AMR profiling was not feasible.

## 5. Conclusions

To summarise the results of our study, ceftiofur will be the most effective antibacterial agent, showing maximum efficacy in most cases against *A. pleuropneumoniae*, *P. multocida*, and *S. suis* isolates. Florfenicol, tulathromycin, and tilmicosin can be highly effective against both *A. pleuropneumoniae* and *P. multocida* infections. For *P. multocida* isolates, amoxicillin, enrofloxacin, and tiamulin have also shown high efficacy, whereas for *A. pleuropneumoniae* isolates, these agents show more moderate efficacy. Tetracyclines showed only moderate sensitivity against both *A. pleuropneumoniae* and *P. multocida* isolates. Enrofloxacin and florfenicol also showed only moderate sensitivity against *S. suis* isolates. The national isolates showed outstanding resistance to the combination sulfamethoxazole-trimethoprim for all three bacteria. However, the high level of resistance to cefquinome in *A*. *pleuropneumoniae* strains, which is of major public health importance, should be highlighted.

## Figures and Tables

**Table 1:** Distribution of MIC values for the clinical isolates of *Actinobacillus pleuropneumoniae* tested and the MIC_50_ and MIC_90_ values. The top row shows the number of pieces, and the bottom row shows the percentage distribution per substance. The red line indicates concentrations that are already resistant to the antibacterial agent, based on CLSI (*) and EUCAST (**) recommendations. +MIC values are relative to the trimethoprim component in the 20:1 sulfamethoxazole/trimethoprim combination.

Antibacterial Agents	Isolates Tested (pcs)	Breakpoints (µg/mL)	Distribution of Bacterial Strains (pcs and %) by Dilution Series (µg/mL)														MIC_50_ (µg/mL)	MIC_90_ (µg/mL)
			128	64	32	16	8	4	2	1	0.5	0.25	0.125	0.06	0.03	0.015		
Amoxicillin	164	≥2 *		36	18	1	3	1		8	9	48	13	13	14		0.25	64
				22.0	11.0	0.6	1.8	0.6		4.9	5.5	29.3	7.9	7.9	8.5			
Ceftiofur	144	≥8 *						6	2	2		3	14	24	32	61	0.03	0.125
								4.1	1.4	1.4		2.1	9.7	16.7	22.2	42.4		
Cefquinome	41	≥0.03 **								2	2	3	6	15	10	3	0.06	0.25
										4.9	4.9	7.3	14.6	36.6	24.4	7.3		
Oxytetracycline	45	≥2 *	4	18	2				1		2	10	1	7			32	64
			8.9	40.0	4.4				2.2		4.4	22.2	2.2	15.6				
Doxycycline	109	-		1	8	23	3	2	8	30	17	8	3	6			1	16
				0.9	7.3	21.1	2.8	1.8	7.3	27.5	15.6	7.3	2.8	5.5				
Tylosin	78	-	6	17	36	4	8		1	1			2	3			32	64
			7.7	21.8	46.2	5.1	10.3		1.3	1.3			2.6	3.8				
Tilmicosin	138	≥32 *	2	3	1	3	43	20	19	29	2	2	2	12			4	8
			1.4	2.2	0.7	2.2	31.2	14.5	13.8	21.0	1.4	1.4	1.4	8.7				
Tylvalosin	58	-	1	37	7		1	1					2	4			64	64
			1.8	63.8	12.1		1.7	1.7					3.4	6.9				
Tulathromycin	139	≥128 *		1	1	23	18	8	4	8	23	27	2	24			0.5	16
				0.7	0.7	16.5	12.9	5.8	2.9	5.8	16.5	19.4	1.4	17.3				
Lincomycin	70	-	5	27	13	15	4							6			32	64
			7.1	38.6	18.6	21.4	5.7							8.6				
Tiamulin	112	≥32 *	9	6	18	23	14	14	10	9	1		2	6			8	64
			8.0	5.4	16.1	20.5	12.5	12.5	8.9	8.0	0.9	0.0	1.8	5.4				
Florfenicol	164	≥8 *			1		1	19	21	54	52	1	3	6	6		1	4
					0.6		0.6	11.6	12.8	32.9	31.7	0.6	1.8	3.7	3.7			
Colistin	88	-			3					8			3		23	51	0.015	1
					3.4					9.1			3.4		26.1	58.0		
Enrofloxacin	141	≥1 *	3	1		2	7	11	25	25	2	2	12	2	9	40	1	4
			2.1	0.7		1.4	5.0	7.8	17.7	17.7	1.4	1.4	8.5	1.4	6.4	28.4		
Sulfamethoxazole/ Trimethoprim+	61	≥0.125 **	5	3		2	7	12	13	5	3	1	4		6		2	64
			8.2	4.9		3.3	11.5	19.7	21.3	8.2	4.9	1.6	6.6		9.8			

**Table 2:** Percentage of susceptibility of clinical isolates of *Actinobacillus pleuropneumoniae* to the antibacterial agents tested, based on CLSI (*) and EUCAST (**) recommendations. +MIC values are relative to the trimethoprim component in the 20:1 sulfamethoxazole/trimethoprim combination.

Antimicrobial Agents	Strains Tested (pcs)	Breakpoints (µg/mL)	Sensitive (%)	Resistant (%)
Amoxicillin	164	≥2 *	64.0	36.0
Ceftiofur	144	≥8 *	100.0	0.0
Cefquinome	41	≥0.03 **	7.3	92.7
Oxytetracycline	45	≥2 *	44.4	55.6
Tilmicosin	138	≥32 *	95.7	4.3
Tulathromycin	139	≥128 *	100.0	0.0
Tiamulin	112	≥32 *	70.5	29.5
Florfenicol	164	≥8 *	98.8	1.2
Enrofloxacin	141	≥1 *	47.5	52.5
Sulfamethoxazole/Trimethoprim+	61	≥0.125 **	9.8	90.8

**Table 3:** MDR profiles detected in *A. pleuropneumoniae* isolates.

Profile Number	Number of Isolates	MDR Profile *
1	12	AM, TIA, ENR
2	8	AM, OTC, TIA
3	2	AM, OTC, TIA, FLO
4	1	AM, OTC, TILM
5	1	OTC, TILM, TIA

* AM = amoxicillin, TIA = tiamulin, ENR = enrofloxacin, OTC = oxytetracycline, FLO = florfenicol, and TILM = tilmicosin.

**Table 4:** Distribution of MIC values for the *Pasteurella multocida* clinical isolates tested and the MIC_50_ and MIC_90_ values. The top row shows the number of pieces, and the bottom row shows the percentage distribution per substance. The red line indicates concentrations that are already resistant to the antibacterial agent, based on CLSI (*) and EUCAST (**) recommendations. +MIC values are relative to the trimethoprim component in the 20:1 sulfamethoxazole/trimethoprim combination.

Antibacterial Agents	Isolates Tested (pcs)	Breakpoints (µg/mL)	Distribution of Bacterial Strains (pcs and %) by Dilution Series (µg/mL)															MIC_50_ (µg/mL)	MIC_90_ (µg/mL)
			256	128	64	32	16	8	4	2	1	0.5	0.25	0.125	0.06	0.03	0.015		
Amoxicillin	65	≥2 *									13	15	21	4	10	2		0.25	1
											20.0	23.1	32.3	6.2	15.4	3.1			
Ceftiofur	63	≥8 *					1				2	1	2	4	7	14	32	0.015	0.125
							1.6				3.2	1.6	3.2	6.3	11.1	22.2	50.8		
Doxycycline	65	≥1 **				2		1	3	5	9	19	7	6	13			0.5	2
						3.1		1.5	4.6	7.7	13.8	29.2	10.8	9.2	20.0				
Tylosin	29	-			2	11	13	3										16	32
					6.9	37.9	44.8	10.3											
Tilmicosin	42	≥32 *				2	1	2	2	11	8	12	4					1	8
						4.8	2.4	4.8	4.8	26.2	19.0	28.6	9.5						
Tulathromycin	59	≥64 *					1		1		1	5	9	17	23		2	0.125	0.5
							1.7		1.7		1.7	8.5	15.3	28.8	39.0		3.4		
Lincomycin	19	-			2	6	6	1		2	2							16	32
					10.5	31.6	31.6	5.3		10.5	10.5								
Tiamulin	44	≥64 **			1	2	13	22	5	1								8	16
					2.3	4.5	29.5	50.0	11.4	2.3									
Florfenicol	65	≥8 *							2	2	11	30	16	1	3			0.5	1
									3.1	3.1	16.9	46.2	24.6	1.5	4.6				
Colistin	25	-								1					8	10	6	0.03	0.06
										4					32	40	24		
Enrofloxacin	41	≥1 *													9	12	20	0.03	0.06
															22.0	29.3	48.8		
Sulfamethoxazole/ Trimethoprim+	54	≥0.125 **	3	2		9	5	4	10	19	2							4	32
			5.6	3.7		16.7	9.3	7.4	18.5	35.2	3.7								

**Table 5:** Percentage of susceptibility of clinical isolates of *Pasteurella multocida* to the antibacterial agents tested based on CLSI (*) and EUCAST (**) recommendations. +MIC values are relative to the trimethoprim component in the 20:1 sulfamethoxazole/trimethoprim combination.

Antimicrobial Agents	Strains Tested (pcs)	Breakpoints (µg/mL)	Sensitive (%)	Resistant (%)
Amoxicillin	65	≥2 *	100.0	0.0
Ceftiofur	63	≥8 *	98.4	1.6
Doxycycline	65	≥1 **	69.2	30.8
Tilmicosin	42	≥32 *	95.2	4.8
Tulathromycin	59	≥64 *	100.0	0.0
Tiamulin	44	≥64 **	97.7	2.3
Florfenicol	65	≥8 *	100.0	0.0
Enrofloxacin	41	≥1 *	100.0	0.0
Sulfamethoxazole/Trimethoprim+	54	≥0.125 **	0.0	100.0

**Table 6:** Distribution of MIC values for the *Streptococcus suis* clinical isolates tested and the MIC_50_ and MIC_90_ values. The top row shows the number of pieces, and the bottom row shows the percentage distribution per substance. The red line indicates concentrations that are already resistant to the antibacterial agent, based on CLSI (*) and EUCAST (**) recommendations. +MIC values are relative to the trimethoprim component in the 20:1 sulfamethoxazole/trimethoprim combination.

Antibacterial Agents	Isolates Tested (pcs)	Breakpoints (µg/mL)	Distribution of Bacterial Strains (pcs and %) by Dilution Series (µg/mL)															MIC_50_ (µg/mL)	MIC_90_ (µg/mL)
			256	128	64	32	16	8	4	2	1	0.5	0.25	0.125	0.06	0.03	0.015		
Amoxicillin	116	-			4	2	4	1	2		4	3	4	12	37	43		0.06	4
					3.4	1.7	3.4	0.9	1.7		3.4	2.6	3.4	10.3	31.9	37.1			
Ceftiofur	117	≥8 *							2	3	8	4	10	3	13	17	57	0.03	1
									1.7	2.6	6.8	3.4	8.5	2.6	11.1	14.5	48.7		
Doxycycline	100	-			3	24	18	6	8	7	2	5	4	8	15			8	32
					3	24	18	6	8	7	2	5	4	8	15				
Tylosin	41	-		15.0		12.0		2.0			3.0	2.0	1.0		6.0			32	128
				36.6		29.3		4.9			7.3	4.9	2.4		14.6				
Tilmicosin	86	-		21	2	11	7	3	1	4	16	3	7	4	7			8	128
				24.4	2.3	12.8	8.1	3.5	1.2	4.7	18.6	3.5	8.1	4.7	8.1				
Tylvalosin	89	-		17	1		14	6	4	7	3	5	8	4	16	2	2	2	128
				19.1	1.1		15.7	6.7	4.5	7.9	3.4	5.6	9.0	4.5	18.0	2.2	2.2		
Lincomycin	40	-		17	3	4	2					2	4		8			32	128
				42.5	7.5	10.0	5.0					5.0	10.0		20.0				
Tiamulin	33	-		8	7	1	1	6	1		1	4	1	1	2			16	128
				24.2	21.2	3.0	3.0	18.2	3.0		3.0	12.1	3.0	3.0	6.1				
Florfenicol	118	≥8 *			8		4	17	24	38	9	5	3	5		5		2	8
					6.8		3.4	14.4	20.3	32.2	7.6	4.2	2.5	4.2		4.2			
Enrofloxacin	70	≥2 *					4	5	1	2	12	12	7	11	6	4	6	0.5	8
							5.7	7.1	1.4	2.9	17.1	17.1	10.0	15.7	8.6	5.7	8.6		
Sulfamethoxazole/ Trimethoprim +	105	≥0.25 **	15	17	2	9	10	13	18	3	2	9	1	6				16	256
			14.3	16.2	1.9	8.6	9.5	12.4	17.1	2.9	1.9	8.6	1.0	5.7					

**Table 7:** Percentage of susceptibility of clinical isolates of *Streptococcus suis* to the antibacterial agents tested, based on CLSI (*) and EUCAST (**) recommendations. +MIC values are relative to the trimethoprim component in the 20:1 sulfamethoxazole/trimethoprim combination.

Antimicrobial Agents	Strains Tested (pcs)	Breakpoints (µg/mL)	Sensitive (%)	Resistant (%)
Ceftiofur	117	≥8 *	100.0	0.0
Florfenicol	118	≥8 *	75.4	24.6
Enrofloxacin	105	≥2 *	82.9	17.1
Sulfamethoxazole/Trimethoprim+	116	≥0.25 **	5.7	94.3

**Table 8:** Number of *A. pleuropneumoniae*, *P. multocida*, and *S. suis* isolates tested, by substance and in total.

Antibacterial Agent	*A. pleuropneumoniae* (pcs)	*P. multocida* (pcs)	*S. suis* (pcs)
Amoxicillin	164	65	116
Ceftiofur	144	63	117
Cefquinome	41	-	-
Oxytetracycline	45	-	-
Doxycycline	109	65	100
Tylosin	78	29	41
Tilmicosin	138	42	86
Tylvalosin	58	-	-
Tulathromycin	139	59	89
Lincomycin	70	19	40
Tiamulin	112	44	33
Florfenicol	164	65	118
Colistin	88	25	-
Enrofloxacin	141	41	70
Sulfamethoxazole: Trimethoprim (20:1)	61	54	105
Total (pcs)	164	65	118

## Data Availability

The data presented in this study are available on request from the corresponding author.

## References

[1] Petri F.A.M., Ferreira G.C., Arruda L.P., Malcher C.S., Storino G.Y., Almeida H.M.d.S., Sonalio K., Silva D.G.D., Oliveira L.G.D. (2023). Associations between Pleurisy and the Main Bacterial Pathogens of the Porcine Respiratory Diseases Complex (PRDC). Animals.

[2] Thacker E.L. (2001). Immunology of the Porcine Respiratory Disease Complex. Vet. Clin. N. Am. Food Anim. Pract..

[3] Chae C. (2016). Porcine Respiratory Disease Complex: Interaction of Vaccination and Porcine Circovirus Type 2, Porcine Reproductive and Respiratory Syndrome Virus, and *Mycoplasma hyopneumoniae*. Vet. J. Lond. Engl. 1997.

[4] Sargeant J.M., Bergevin M.D., Churchill K., Dawkins K., Deb B., Dunn J., Hu D., Moody C., O’Connor A.M., O’Sullivan T.L. (2019). A Systematic Review of the Efficacy of Antibiotics for the Prevention of Swine Respiratory Disease. Anim. Health Res. Rev..

[5] Saade G., Deblanc C., Bougon J., Marois-Créhan C., Fablet C., Auray G., Belloc C., Leblanc-Maridor M., Gagnon C.A., Zhu J. (2020). Coinfections and Their Molecular Consequences in the Porcine Respiratory Tract. Vet. Res..

[6] El Garch F., de Jong A., Simjee S., Moyaert H., Klein U., Ludwig C., Marion H., Haag-Diergarten S., Richard-Mazet A., Thomas V. (2016). Monitoring of Antimicrobial Susceptibility of Respiratory Tract Pathogens Isolated from Diseased Cattle and Pigs across Europe, 2009–2012: VetPath Results. Vet. Microbiol..

[7] Antimicrobial Wild Type Distributions of Microorganisms, EUCAST. https://mic.eucast.org/search/?search%5Bmethod%5D=mic&search%5Bantibiotic%5D=-1&search%5Bspecies%5D=-1&search%5Bdisk_content%5D=-1&search%5Blimit%5D=50.

[8] Holmer I., Salomonsen C.M., Jorsal S.E., Astrup L.B., Jensen V.F., Høg B.B., Pedersen K. (2019). Antibiotic Resistance in Porcine Pathogenic Bacteria and Relation to Antibiotic Usage. BMC Vet. Res..

[9] Mader R., Muñoz Madero C., Aasmäe B., Bourély C., Broens E.M., Busani L., Callens B., Collineau L., Crespo-Robledo P., Damborg P. (2022). Review and Analysis of National Monitoring Systems for Antimicrobial Resistance in Animal Bacterial Pathogens in Europe: A Basis for the Development of the European Antimicrobial Resistance Surveillance Network in Veterinary Medicine (EARS-Vet). Front. Microbiol..

[10] Toutain P.-L., Pelligand L., Lees P., Bousquet-Mélou A., Ferran A.A., Turnidge J.D. (2021). The Pharmacokinetic/Pharmacodynamic Paradigm for Antimicrobial Drugs in Veterinary Medicine: Recent Advances and Critical Appraisal. J. Vet. Pharmacol. Ther..

[11] Kowalska-Krochmal B., Dudek-Wicher R. (2021). The Minimum Inhibitory Concentration of Antibiotics: Methods, Interpretation, Clinical Relevance. Pathogens.

[12] Kardos G., Sárközi R., Laczkó L., Marton S., Makrai L., Bányai K., Fodor L. (2022). Genetic Diversity of Actinobacillus Pleuropneumoniae Serovars in Hungary. Vet. Sci..

[13] Burch D.G.S., Sperling D. (2018). Amoxicillin—Current Use in Swine Medicine. J. Vet. Pharmacol. Ther..

[14] European Medicines Agency Categorisation of Antibiotics in the European Union 2019. https://www.ema.europa.eu/en/documents/report/categorisation-antibiotics-european-union-answer-request-european-commission-updating-scientific_en.pdf.

[15] Chander Y., Oliveira S., Goyal S.M. (2011). Characterisation of Ceftiofur Resistance in Swine Bacterial Pathogens. Vet. J..

[16] Zhang L., Wu X., Huang Z., Zhang N., Wu Y., Cai Q., Shen X., Ding H. (2018). Pharmacokinetic/Pharmacodynamic Assessment of Cefquinome against *Actinobacillus Pleuropneumoniae* in a Piglet Tissue Cage Infection Model. Vet. Microbiol..

[17] Zhang L., Xie H., Wang H., Ding H., Zhang G., Hu J. (2021). Kill Rate and Evaluation of Ex Vivo PK/PD Integration of Cefquinome against *Actinobacillus Pleuropneumoniae*. Front. Vet. Sci..

[18] Tóth A.G., Csabai I., Maróti G., Jerzsele Á., Dubecz A., Patai Á.V., Judge M.F., Nagy S.Á., Makrai L., Bányai K. (2020). A Glimpse of Antimicrobial Resistance Gene Diversity in Kefir and Yoghurt. Sci. Rep..

[19] Cheng D., Feng Y., Liu Y., Xue J., Li Z. (2019). Dynamics of Oxytetracycline, Sulfamerazine, and Ciprofloxacin and Related Antibiotic Resistance Genes during Swine Manure Composting. J. Environ. Manag..

[20] Mazurek J., Bok E., Stosik M., Baldy-Chudzik K. (2015). Antimicrobial Resistance in Commensal Escherichia Coli from Pigs during Metaphylactic Trimethoprim and Sulfamethoxazole Treatment and in the Post-Exposure Period. Int. J. Environ. Res. Public Health.

[21] Yang F., Liu H.W., Li M., Ding H.Z., Huang X.H., Zeng Z.L. (2012). Use of a Monte Carlo Analysis within a Physiologically Based Pharmacokinetic Model to Predict Doxycycline Residue Withdrawal Time in Edible Tissues in Swine. Food Addit. Contam. Part A.

[22] Dorey L., Pelligand L., Cheng Z., Lees P. (2017). Pharmacokinetic/Pharmacodynamic Integration and Modelling of Florfenicol for the Pig Pneumonia Pathogens *Actinobacillus Pleuropneumoniae* and *Pasteurella Multocida*. PLoS ONE.

[23] Xiong J., Zhu Q., Yang S., Zhao Y., Cui L., Zhuang F., Qiu Y., Cao J. (2019). Comparison of Pharmacokinetics of Tilmicosin in Healthy Pigs and Pigs Experimentally Infected with *Actinobacillus Pleuropneumoniae*. N. Z. Vet. J..

[24] Huang L., Zhang H., Li M., Ahmad I., Wang Y., Yuan Z. (2018). Pharmacokinetic-Pharmacodynamic Modeling of Tylosin against *Streptococcus suis* in Pigs. BMC Vet. Res..

[25] Zhou Q., Zhang G., Wang Q., Liu W., Huang Y., Yu P., Li Y., Ding H., Fang B. (2017). Pharmacokinetic/Pharmacodynamic Modeling of Tulathromycin against Pasteurella Multocida in a Porcine Tissue Cage Model. Front. Pharmacol..

[26] Bladek T., Posyniak A., Jablonski A., Gajda A. (2015). Pharmacokinetics of Tulathromycin in Edible Tissues of Healthy and Experimentally Infected Pigs with *Actinobacillus Pleuropneumoniae*. Food Addit. Contam. Part A.

[27] Nielsen P., Gyrd-Hansen N. (1998). Bioavailability of Spiramycin and Lincomycin after Oral Administration to Fed and Fasted Pigs. J. Vet. Pharmacol. Ther..

[28] Pallarés F.J., Lasa C., Roozen M., Ramis G. (2015). Use of Tylvalosin in the Control of Porcine Enzootic Pneumonia. Vet. Rec. Open.

[29] Albert E., Sipos R., Perreten V., Tóth Á., Ungvári E., Papp M., Dán Á., Biksi I. (2023). High Prevalence of Livestock-Associated Methicillin-Resistant *Staphylococcus aureus* in Hungarian Pig Farms and Genomic Evidence for the Spillover of the Pathogen to Humans. Transbound. Emerg. Dis..

[30] Somogyi Z., Mag P., Kovács D., Kerek Á., Szabó P., Makrai L., Jerzsele Á. (2022). Synovial and Systemic Pharmacokinetics of Florfenicol and PK/PD Integration against *Streptococcus suis* in Pigs. Pharmaceutics.

[31] Somogyi Z., Mag P., Simon R., Kerek Á., Szabó P., Albert E., Biksi I., Jerzsele Á. (2023). Pharmacokinetics and Pharmacodynamics of Florfenicol in Plasma and Synovial Fluid of Pigs at a Dose of 30 Mg/Kgbw Following Intramuscular Administration. Antibiotics.

[32] Grégoire N., Aranzana-Climent V., Magréault S., Marchand S., Couet W. (2017). Clinical Pharmacokinetics and Pharmacodynamics of Colistin. Clin. Pharmacokinet..

[33] Rhouma M., Beaudry F., Thériault W., Letellier A. (2016). Colistin in Pig Production: Chemistry, Mechanism of Antibacterial Action, Microbial Resistance Emergence, and One Health Perspectives. Front. Microbiol..

[34] Rhouma M., Beaudry F., Thériault W., Bergeron N., Beauchamp G., Laurent-Lewandowski S., Fairbrother J.M., Letellier A. (2016). In Vivo Therapeutic Efficacy and Pharmacokinetics of Colistin Sulfate in an Experimental Model of Enterotoxigenic Escherichia Coli Infection in Weaned Pigs. Vet. Res..

[35] Chen T., Xie G., Mi J., Wen X., Cao Z., Ma B., Zou Y., Zhang N., Wang Y., Liao X. (2022). Recovery of the Structure and Function of the Pig Manure Bacterial Community after Enrofloxacin Exposure. Microbiol. Spectr..

[36] De Smet J., Boyen F., Croubels S., Rasschaert G., Haesebrouck F., Temmerman R., Rutjens S., De Backer P., Devreese M. (2020). The Impact of Therapeutic-Dose Induced Intestinal Enrofloxacin Concentrations in Healthy Pigs on Fecal *Escherichia coli* Populations. BMC Vet. Res..

[37] González-Fandos E., Martínez-Laorden A., Abad-Fau A., Sevilla E., Bolea R., Serrano M.J., Mitjana O., Bonastre C., Laborda A., Falceto M.V. (2022). Effect of Intramuscularly Administered Oxytetracycline or Enrofloxacin on Vancomycin-Resistant Enterococci, Extended Spectrum Beta-Lactamase- and Carbapenemase-Producing Enterobacteriaceae in Pigs. Animals.

[38] Kaspersen H., Urdahl A.M., Grøntvedt C.A., Gulliksen S.M., Tesfamichael B., Slettemeås J.S., Norström M., Sekse C. (2020). *Actinobacillus pleuropneumoniae* Eradication with Enrofloxacin May Lead to Dissemination and Long-Term Persistence of Quinolone Resistant Escherichia Coli in Pig Herds. Antibiotics.

[39] Matamoros V., Casas M.E., Pastor E., Tadić Đ., Cañameras N., Carazo N., Bayona J.M. (2022). Effects of Tetracycline, Sulfonamide, Fluoroquinolone, and Lincosamide Load in Pig Slurry on Lettuce: Agricultural and Human Health Implications. Environ. Res..

[40] Yang B., Li X.D., Chen X., Hong J., Liu C., Zheng J.P., Ou Z.Y., Yu D.J. (2022). PK/PD Modelling of Enrofloxacin against *Glaesserella parasuis* Infection in Pigs. J. Vet. Pharmacol. Ther..

[41] CLSI (2020). Performance Standards for Antimicrobial Disk Dilution Susceptibility Tests for Bacteria Isolated from Animals.

[42] 128/2009. (X. 6.) FVM Rendelet az Állatgyógyászati Termékekről. https://net.jogtar.hu/jogszabaly?docid=A0900128.FVM&searchUrl=%2Fgyorskereso%3Fkeyword%3Dfvm%2520128%2F2009.

[43] Committee S. (2001). Directive 2001/82/EC of the European Parliament and of the Council of 6 November 2001 on the Community Code Relating to Veterinary Medicinal Products. Off. J. L.

[44] European Union (2018). Regulation (EU) 2019/6 of the European Parliament and of the Council of 11 December 2018 on Veterinary Medicinal Products and Repealing Directive 2001/82/EC (Text with EEA Relevance). Off. J. Eur. Union.

[45] Portis E. (2013). Antimicrobial Susceptibility of Porcine *Pasteurella multocida*, *Streptococcus suis*, and *Actinobacillus pleuropneumoniae* from the United States and Canada, 2001 to 2010. J. Swine Health Prod..

[46] Kucerova Z., Hradecka H., Nechvatalova K., Nedbalcova K. (2011). Antimicrobial Susceptibility of *Actinobacillus pleuropneumoniae* Isolates from Clinical Outbreaks of Porcine Respiratory Diseases. Vet. Microbiol..

[47] Sweeney M.T. (2017). Antimicrobial Susceptibility of *Actinobacillus pleuropneumoniae*, *Pasteurella multocida*, *Streptococcus suis*, and *Bordetella bronchiseptica* Isolated from Pigs in the United States and Canada, 2011 to 2015. J. Swine Health Prod..

[48] Vilaró A., Novell E., Enrique-Tarancón V., Balielles J., Vilalta C., Martinez S., Fraile Sauce L.J. (2020). Antimicrobial Susceptibility Pattern of Porcine Respiratory Bacteria in Spain. Antibiotics.

[49] Gutiérrez-Martín C.B., Blanco N.G.D., Blanco M., Navas J., Rodríguez-Ferri E.F. (2006). Changes in Antimicrobial Susceptibility of *Actinobacillus pleuropneumoniae* Isolated from Pigs in Spain during the Last Decade. Vet. Microbiol..

[50] Yuan Y., An B., Xie S., Qu W., Hao H., Huang L., Luo W., Liang J., Peng D. (2023). The Dose Regimen Formulation of Doxycycline Hydrochloride and Florfenicol Injection Based on Ex Vivo Pharmacokinetic-Pharmacodynamic Modeling against the *Actinobacillus pleuropneumoniae* in Pigs. Anim. Dis..

[51] Chang C.-F., Chang L.-C., Chang Y.-F., Chen M., Chiang T.-S. (2002). Antimicrobial Susceptibility of *Actinobacillus pleuropneumoniae*, *Escherichia coli*, and *Salmonella choleraesuis* Recovered from Taiwanese Swine. J. Vet. Diagn. Investig..

[52] Matter D., Rossano A., Limat S., Vorlet-Fawer L., Brodard I., Perreten V. (2007). Antimicrobial Resistance Profile of *Actinobacillus pleuropneumoniae* and *Actinobacillus porcitonsillarum*. Vet. Microbiol..

[53] Oh Y.-H., Moon D.-C., Lee Y.J., Hyun B.-H., Lim S.-K. (2018). Antimicrobial Resistance of Pasteurella Multocida Strains Isolated from Pigs between 2010 and 2016. Vet. Rec. Open.

[54] Cuevas I., Carbonero A., Cano D., García-Bocanegra I., Amaro M.Á., Borge C. (2020). Antimicrobial Resistance of Pasteurella Multocida Type B Isolates Associated with Acute Septicemia in Pigs and Cattle in Spain. BMC Vet. Res..

[55] Wisselink H.J., Veldman K.T., Van den Eede C., Salmon S.A., Mevius D.J. (2006). Quantitative Susceptibility of *Streptococcus suis* Strains Isolated from Diseased Pigs in Seven European Countries to Antimicrobial Agents Licenced in Veterinary Medicine. Vet. Microbiol..

[56] Hernandez-Garcia J., Wang J., Restif O., Holmes M.A., Mather A.E., Weinert L.A., Wileman T.M., Thomson J.R., Langford P.R., Wren B.W. (2017). Patterns of Antimicrobial Resistance in Streptococcus Suis Isolates from Pigs with or without Streptococcal Disease in England between 2009 and 2014. Vet. Microbiol..

[57] van Hout J., Heuvelink A., Gonggrijp M. (2016). Monitoring of Antimicrobial Susceptibility of Streptococcus Suis in the Netherlands, 2013–2015. Vet. Microbiol..

[58] Magiorakos A.-P., Srinivasan A., Carey R.B., Carmeli Y., Falagas M.E., Giske C.G., Harbarth S., Hindler J.F., Kahlmeter G., Olsson-Liljequist B. (2012). Multidrug-Resistant, Extensively Drug-Resistant and Pandrug-Resistant Bacteria: An International Expert Proposal for Interim Standard Definitions for Acquired Resistance. Clin. Microbiol. Infect..

